# Correction to “S100A9 Aggravates Cardiac Fibrosis by TLR4/PGC‐1α Mediated Macrophage‐Myofibroblast Crosstalk”

**DOI:** 10.1002/jev2.70360

**Published:** 2026-08-29

Zhao, Y., Y. Zhang, Q. Yuan, et al. 2026. “S100A9 Aggravates Cardiac Fibrosis by TLR4/PGC‐1α Mediated Macrophage‐Myofibroblast Crosstalk.” *Journal of Extracellular Vesicles* 15, no. 7: e70338. https://doi.org/10.1002/jev2.70338

In the originally published article, statistical significance markers were missing from Figures 1Q and 6G. The correct figures appear below. The online version of the article has been updated.

We apologize for this error.

**FIGURE 1:**
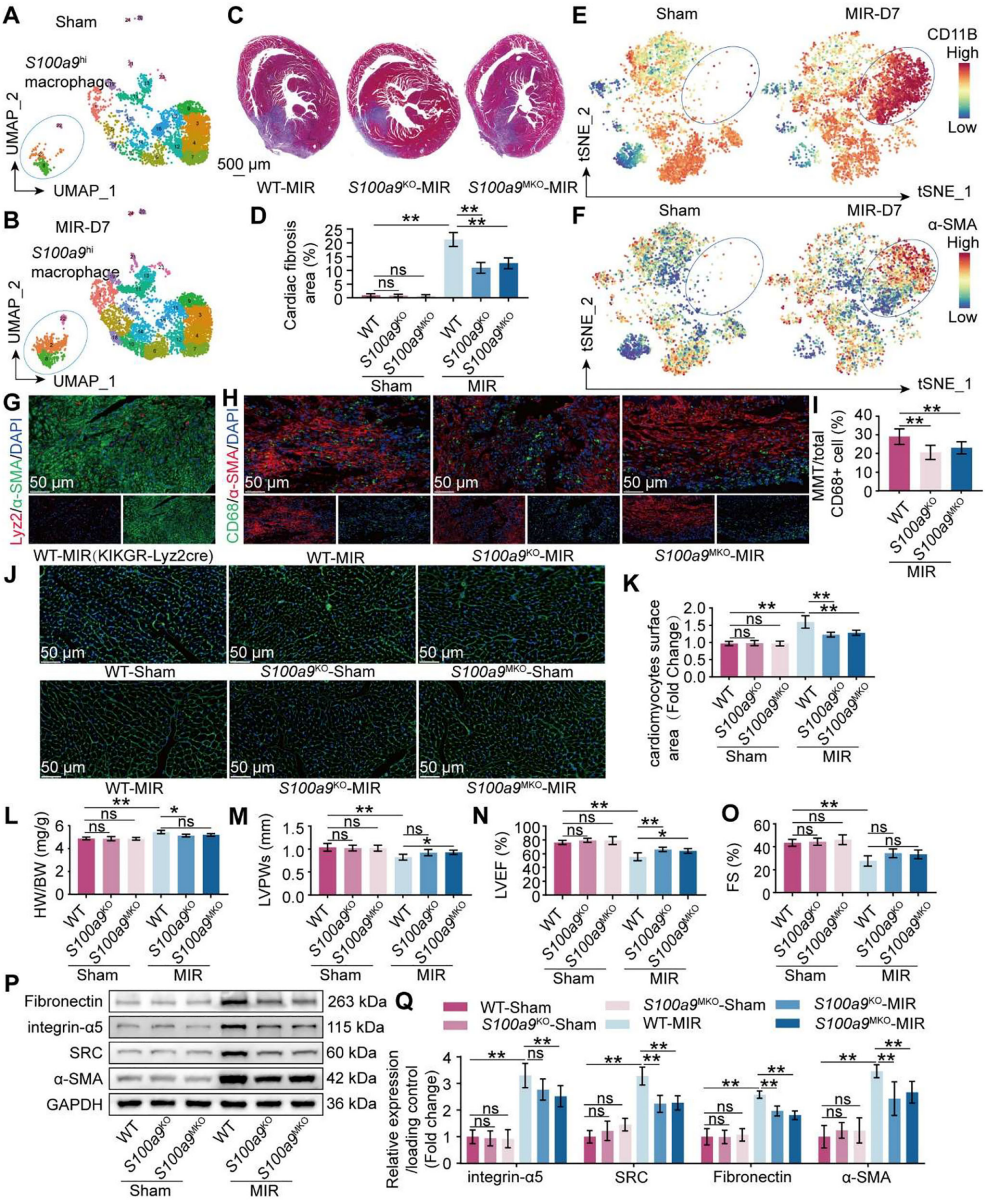
S100A9 promotes macrophage‐to‐myofibroblast transition (MMT) after MI and exacerbates myocardial fibrosis. (A–B) UMAP plots showing the single‐cell transcriptomic landscape of cardiac macrophages from Sham mice and mice at day 7 post–MIR. *n* = 9. (C–D) Masson staining showing cardiac fibrosis at day 7 after MIR, with corresponding quantitative analysis. *n* = 5. (E–F) t‐SNE plots showing MMT levels in cardiac macrophages from Sham and day‐7 MIR mice. *n* = 9. (G) Detection of α‐SMA and endogenously red‐fluorescent macrophages in heart tissue by immunofluorescence. *n* = 3. (H–I) Immunofluorescence staining of α‐SMA and CD68 in heart tissue, with relative quantification. *n* = 5. (J–K) WGA staining showing cardiomyocyte cross‐sectional area with normalized quantification. *n* = 5. (L) Heart weight/body weight ratios and correlative analyses in each group. *n* = 5. (M–O) Echocardiographic assessment of cardiac function, including LVPWs, LVEF, and FS, with quantitative analysis. *n* = 5. (P–Q) Western blot analysis of protein expression in mouse heart tissue with normalized quantification. *n* = 5. ns, not significant; *, *p* < 0.05; **, *p* < 0.01.

**FIGURE 6:**
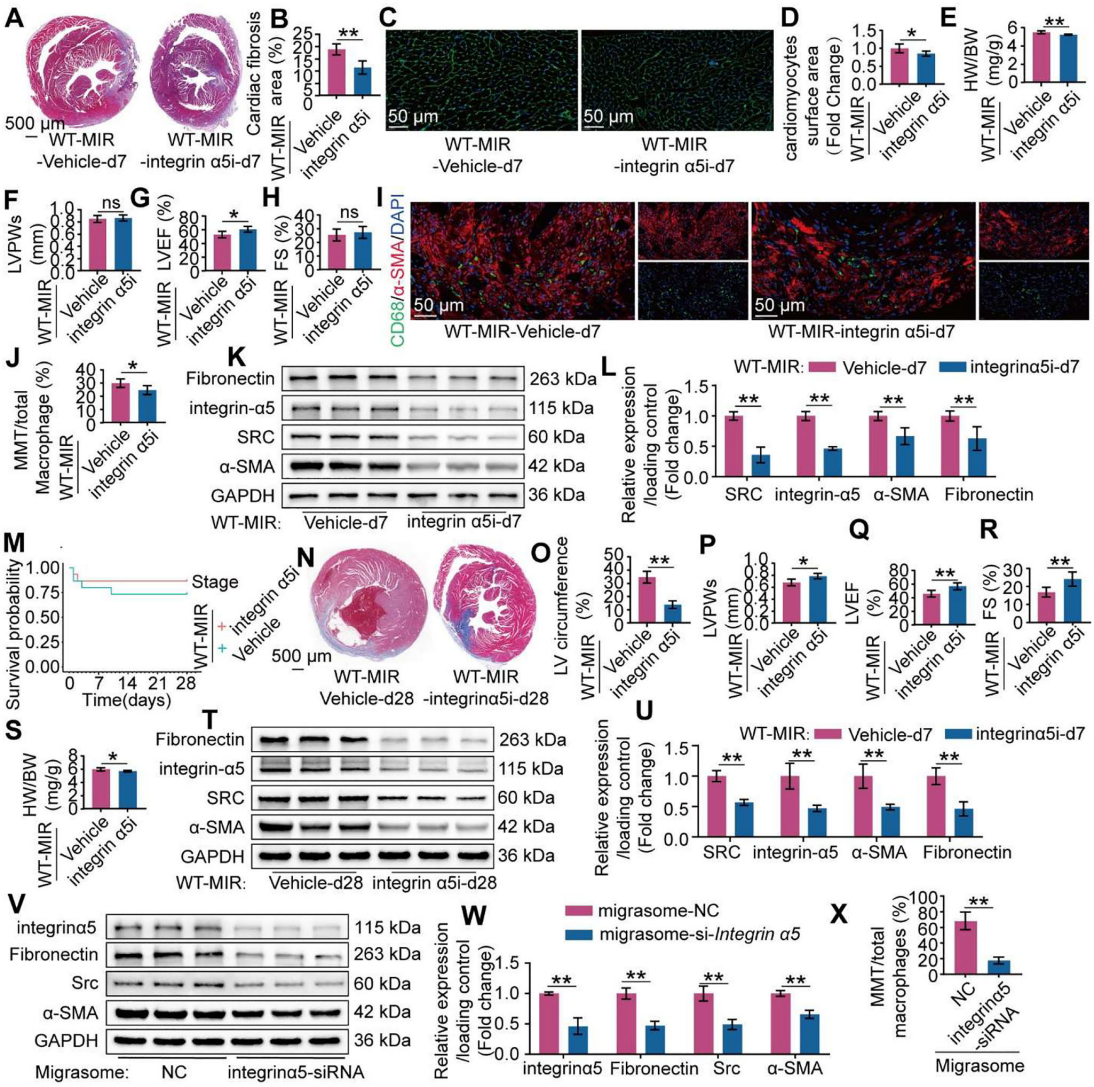
Fibroblast‐derived migrasomes promote MMT through the integrin α5/Src signaling pathway. (A–B) Masson staining showing cardiac fibrosis in mice at Day 7 post‐MIR, with corresponding quantitative analysis. *n* = 5. (C–D) WGA staining showing cardiomyocyte cross‐sectional area with normalized quantification. *n* = 5. (E) Heart weight/body weight ratios and correlative analyses in each experimental group. *n* = 5. (F–H) Echocardiographic assessment of cardiac function, including LVPWs, LVEF, and FS, with quantitative analysis. *n* = 5. (I–J) Immunofluorescence staining of *α*‐SMA and CD68 in heart tissue, with relative quantification. *n* = 5. (K–L) Western blot analysis of protein expression in mouse heart tissue with normalized quantification. *n* = 5. (M) Survival rates of mice following MIR and treatment with integrin *α*5 inhibitor over a 28‐day period. *n* = 15. (N–O) Masson staining showing cardiac fibrosis in mice at day 28 post‐MIR, with corresponding quantitative analysis. *n* = 5. (P) Heart weight/body weight ratios and correlative analyses in each experimental group. *n* = 5. (Q–S) Echocardiographic assessment of cardiac function, including LVPWs, LVEF, and FS, with quantitative analysis. *n* = 5. (T–U) Western blot analysis of protein expression in mouse heart tissue with normalized quantification. *n* = 5. (V–W) MMT signaling expression in macrophages upon migrasome stimulation after integrin *α*5 knockdown. *n* = 5. (X) Flow cytometry analysis of macrophages knockdown integrin *α*5 co‐expressing *α*‐SMA and F4/80 which co‐cultured with migrasome. *n* = 5. ns, not significant; *, *p <* 0.05; **, *p <* 0.01.

